# N-terminal pro-BNP predicts mortality better than procalcitonin in abdominal severe sepsis and septic shock

**DOI:** 10.1186/cc9695

**Published:** 2011-03-11

**Authors:** N Ruiz-Vera, MJ Antolino-Martinez, A Gonzalez-Lisorge, C Garcia-Palenciano, T Sansano-Sanchez, F Acosta-Villegas

**Affiliations:** 1 University Hospital, Murcia, Spain; 2 University Hospital 'Virgen de la Arrixaca', Murcia, Spain

## Introduction

N-terminal pro-BNP (pBNP) could be useful to predict outcome in severe sepsis. We have conducted a study to compare pBNP and procalcitonin (PCT) in the setting of abdominal severe sepsis or septic shock.

## Methods

We performed a prospective study of 51 consecutive patients with abdominal severe sepsis or septic shock. Age, gender, APACHE II score at admission, in-unit survival, presence of septic shock and serum PCT and pBNP levels during 4 days after admission were determined. Statistics: chi-square test, Student's *t *test, Mann-Whitney's test for samples without normal distribution and Cox's logistic regression. *P *< 0.05 was considered statistically significant.

## Results

The mean APACHE II score at admission was 20.52 ± 5.07. This value was found to be significantly higher in nonsurvivors (18.38 ± 4.56 vs. 24.00 ± 4.03, *P *< 0.05). Values of pBNP were significantly higher in nonsurvivors from the first day of the study. PCT levels were higher in nonsurvivors, but only reached statistically significance on day 2 (Table 1). These results were not found to be influenced by age, gender or presence of shock in multivariate analysis.

**Table 1:** Values of pBNP and PCT during the study period

	Day 1	Day 2	Day 3	Day 4
**pBNP (median and Q25 to 75) (pg/ml)**				
Survivors	2,256.50 (1,071 to 2,832)*	1,598.00 (1,412.75 to 3,918.25)	2,102.50 (1,323.50 to 6,166.50)	1,809,00 (939.25 to 5,495.75)
Nonsurvivors	4,090.50 (3,064 to 32,147.75)	8,994,00 (4,911 to 27,860.25)	9,528.00 (3,747.75 to 25,793.2)	5,498,00 (1542 to 19,947.25)
**PCT (mean ± SD) (ng/ml)**				
Survivors	10.13 ± 13.02	11.68 ± 18.29*	12.75 ± 23.16	11.90 ± 24.24
Nonsurvivors	19.81 ± 23.32	25.91 ± 26.87	26.82 ± 26.46	9.89 ± 8.87

*P < 0.05.

## Conclusions

Our results shown that pBNP could be more useful than PCT to discriminate the patients with abdominal severe sepsis and worse outcome.
